# Development of a Comprehensive Approach for the Early Diagnosis of Geriatric Syndromes in General Practice

**DOI:** 10.3389/fmed.2015.00078

**Published:** 2015-11-18

**Authors:** Nicolas Senn, Stéfanie Monod

**Affiliations:** ^1^Department of Ambulatory Care and Community Medicine, University of Lausanne, Lausanne, Switzerland; ^2^Public Health Office Canton de Vaud, Lausanne, Switzerland

**Keywords:** geriatric syndromes, primary care, general practice, diagnostic, management

## Abstract

According to demographic projections, a significant increase in the proportion of the elderly population is anticipated worldwide. This aging of the population will lead to an increase in the prevalence of chronic diseases and functional impairment. This expected increase will result in growing use of the health care system that societies are largely unprepared to address. General practitioners (GPs) are at the front line of this huge epidemiological challenge, but appropriate tools to diagnose and manage elderly patients in routine general practice are lacking. Indeed, while primary prevention and the management of common chronic diseases, such as hypertension, diabetes, or cardiac ischemic diseases, are routinely and mostly adequately performed in primary care, the management of geriatric syndromes is often incomplete. In order to address these shortcomings, this theoretical work aims to first develop, based on the best available evidence, a brief assessment tool (BAT) specifically designed for geriatric syndromes identification in general practice and, second, to propose a conceptual framework for the management of elderly patients in general practice that integrates the BAT instrument into the usual care of GPs. To avoid proposing unachievable goals for the care of elderly patients in general practice (for example, performing all the best screening tools for geriatric conditions identification and care), this work proposes an innovative way to combine geriatric assessment with the management of common chronic diseases.

## Introduction

According to demographic projections, a significant increase in the proportion of the elderly population is anticipated worldwide. By 2030, in industrialized countries, the proportion of persons over the age of 65 years will increase from 15% at present to 22% (1). The population over the age of 80 years will grow the fastest. This aging of the population will lead to an increase in the prevalence of chronic diseases and functional impairment (2).

Improving the prevention of chronic diseases in children and adults and reducing functional decline in elderly persons are therefore urgent. General practitioners (GPs) are in the front line of this huge epidemiological challenge. Interventions should therefore focus on the prevention (primary, secondary, and tertiary) of health problems, especially those most frequently associated with functional impairment.

Though they are also “chronic,” geriatric syndromes differ from “chronic diseases” in the sense that they have multiple underlying factors and involve multiple organ systems (3). Tinetti and colleagues proposed the following definition: “*multifactorial health conditions that occur when the accumulated effects of impairments in multiple systems render [an older] person vulnerable to situational challenges*” (4). Overall, their impact on quality of life (QOL) and disability is considerable. However, if recognized early, adapted preventative measures can be initiated to reduce part of the burden, (5, 6) notably by decreasing the risk of hospitalization and institutionalization and improving the QOL of elderly patients. The comprehensive geriatric assessment and management of geriatric conditions, in particular, have proven efficient to prevent functional decline and institutionalization (6, 7).

While primary prevention and management of common chronic diseases, such as hypertension, diabetes, or cardiac ischemic diseases, are routinely and mostly adequately performed in general practice, the management of geriatric syndromes is often incomplete (8–10). In one study measuring the adherence of GPs to recommendations using quality indicators, it was observed that only half of elderly patients had routine cognitive testing and only one quarter were screened annually for falls (11). In another recent study, screening was especially poor for dementia, depression, and osteoporosis (8). Several reasons may explain this situation. First, numerous GPs are not fully aware of geriatric syndromes and their functional consequences. As a result, interventions to improve QOL or reduce the risks of morbidity and institutionalization in elderly persons are often unknown to GPs. Second, the relevance of an early diagnosis of geriatric problems is often questioned because it is widely believed that they cannot be treated or even stabilized. Finally, GPs often lack the time necessary to perform a comprehensive geriatric evaluation, or at least a screening for geriatric syndromes.

Recent literature has described numerous tools for the identification of geriatric syndromes, but few were specifically designed for general practice, and even those that exist suffer important limitations (12–14). Among the few existing comprehensive tools, the Geriatric Assessment Tool developed by Mann et al. in Austria and the SHARE frailty index could be cited (15, 16). While long to administer (the first one took more than half an hour to administer in a pilot study), the other main limitation is that these tools are designed to be administered as a single intervention, without taking into account constraints common in general practice, such as limited time to perform the assessment and their integration with usual care and the management of other concomitant comorbidities. There is a need to rethink the way medical consultations are performed for elderly patients in general practice and to develop new conceptual frameworks.

This work aims to, first, discuss relevant geriatric syndromes, second, to develop, based on a literature review and best available evidence, a brief assessment tool (BAT) specifically designed for GPs, and, third, to propose a conceptual framework for the management of elderly patients in general practice that integrates the BAT into the usual care of GPs.

## Methods

The present work is a theoretical development performed by both a GP and a geriatrician with the aim of developing the BAT for the early diagnosis of geriatric syndromes in general practice. Based on this evidence, a conceptual framework for its integration into the daily practice of GPs will be proposed. Identification of geriatric syndromes and screening tests relevant in PC was based on a scoping literature review. The following research terms (MESH terms) were used alone and in sensible combinations to select relevant publications for the identification of geriatric syndromes and their clinical tests: elderly, old, geriatric syndromes, geriatric conditions, frailty, primary care, general practice, family medicine, quality of life, screening instruments, screening tests, screening tool, early diagnosis, test, functional impairment, functional decline, cognitive impairment, affective disorder, depression, mood disorder, gait and balance disorder, falls, sleep problems, visual and hearing impairment, visual and hearing loss, pain, nutrition, osteoporosis, urinary incontinence, activity of day life (ADL), instrumental ADL (IADL), interpersonal relationship, safety, and elder mistreatment, finances.

### Important Geriatric Syndromes and Screening Tests

An initial listing of potential syndromes was made based on a literature review. Five inclusion criteria were used to select geriatric syndromes: (1) the syndrome is prevalent in the elderly population; (2) a relationship exists between the syndrome and functional decline and/or QOL in the elderly population; (3) the syndrome is clinically relevant in general practice (i.e., it has a certain level of impact on morbidity/mortality); (4) screening is feasible in general practice (according to several studies, tests best suited for GPs should last between 2 and 5 min. (17–19)); and (5) management options to prevent functional decline or improve QOL exist for the syndrome. Each of the syndromes was then rated by the investigators for all five criteria on a Likert scale from 0 (not important at all) to 3 (very important) in order to describe their specific relevance. This semi-qualitative approach allows an integrated assessment of the different syndromes and provides a pragmatic approach to select the most relevant ones.

The second part of the work was aimed at identifying tests for geriatric syndromes. We defined three criteria that needed to be present to retain a test: (1) the test was validated in elderly patients; (2) performances of the tests were assessed and considered acceptable; (3) the test was developed for general practice or at least is feasible in general practice (time/infrastructures).

A BAT instrument was developed based on the geriatric syndromes and best corresponding tests were retained.

### Development of a Conceptual Framework to Integrate the BAT into Routine General Practice

Following the initial identification of geriatric syndromes and tests, a conceptual framework within which the BAT should be performed in general practice was elaborated. This framework was developed by taking into consideration the following questions: (1) When should a BAT be performed in general practice? (2) For which patients? and (3) How should a BAT be integrated into GPs practices? To answer this last question, the authors took into consideration the following specificities of the general practice context: (1) GPs have a comprehensive approach to patients (taking into account the patient’s clinical and social contexts) and are, thus, able not only to make decisions on the management of single diseases but also to adopt a more global and individualized approach to complex situations; (2) by managing patients on the long term (longitudinality), GPs are the health care providers best able to anticipate functional decline; (3) the usual time for one consultation is short, but the patient may be seen frequently; (4) during routine consultations, GPs have to balance the current health problems of the patient (including medication prescription) into proactive interventions such as prevention or, in this case, a BAT for geriatric syndromes screening.

## Results

### Relevant Geriatric Syndromes and Tests to be Used in GP Practices

The five conditions most commonly considered as geriatric syndromes are cognitive impairment, pressure ulcers, incontinence, falls, functional decline, and delirium (3, 20–22). Additionally, affective disorders, visual and hearing impairment, and malnutrition are often cited (22, 23). To a lesser extent, eating and feeding problems, sleeping problems, dizziness and syncope, self-neglect and elder abuse have also been classified as geriatric syndromes (6, 24–26).

For the present work, eight geriatric syndromes were retained: Cognitive impairment, mood disorder, urinary incontinence, malnutrition, gait and balance impairment and falls, osteoporosis, hearing loss, and visual impairment. The Data Sheet S1 in Supplementary Material provides a detailed references narrative report that justifies the selection geriatric syndromes. Table 1 summarize these syndromes with their respective definition, prevalence rates in community-dwelling elderly persons, as well as their impact on functional independence and relevance for screening in general practice. The following syndromes were not retained either because the pre-specified criteria were not met (see METHODS): pressure ulcers, sleeping problems, dizziness and syncope, self-neglect, and elder abuse. Delirium was not retained because it is often an acute event that is rarely seen in GP practices.

**Table 1:** **Definition and prevalence of the eight geriatric syndromes and relevance of screening in general practice [legend: +++ = highly relevant, (+) = not very relevant]**.

Name of syndrome	Definition/criteria	Prevalence	Relevance of screening for different geriatric syndromes in primary care					Reference
			Associated with functional dependency	Highly prevalent in PC	Clinically relevant (impact on morbidity/mortality)	Screening feasible in PC	Supportive management options are available	
Cognitive impairment	Syndrome due to disease of the brain, usually of a chronic or progressive nature, in which there is disturbance of multiple higher cortical functions, including memory, thinking, orientation, comprehension, calculation, learning capacity, language, and judgment. Consciousness is not clouded. The impairments of cognitive function are commonly accompanied, and occasionally preceded, by deterioration in emotional control, social behavior, or motivation. This syndrome occurs in Alzheimer disease, in cerebrovascular disease, and in other conditions primarily or secondarily affecting the brain	3–4% in 70–74 years >10% in >80 years	++(+)	++(+)	+++	+++	++	ICD-10 (27)
Mood disorder/depression	Change in affect or mood to depression (with or without associated anxiety) or to elation. The mood change is usually accompanied by a change in the overall level of activity; most of the other symptoms are either secondary to, or easily understood in the context of, the change in mood and activity. (ICD-10)	8–15% in >65 years (community)	++	++(+)	++	++(+)	++(+)	ICD-10 (28, 29)
Urinary incontinence	The loss of bladder control	30% (community-dwelling)	+(+)	++	+(+)	+++	++(+)	(30, 31)
		9–39% daily UI women >60 years	
		2–11% daily UI in older men	
Gait and balance impairment/falls	Gait and balance impairment resulting in an increase risk of fall over time	35–45% of population >65 years old fall at least once/year	+++	+++	++(+)	+++	++(+)	(32)
Visual impairment	Blindness: inability to count fingers at a distance of 10 feet (~3 m) and is labeled 10/200 (3/60 or 0.05), meaning that a “Normal” person would be able to count these fingers at a distance of 200 feet. This vision does no longer allow to read, regardless of the font size. There are different degrees of severity ranging from residual vision better eye corrected to complete blindness with no light perception	Visual impairment (difficulties to read newspapers)	++(+)	++(+)	+(+)	+++	++	(33)
	Low vision: acuity between 20/60 (6/18 or 0.32) and 10/200 (3/60 or 0.05). This is a significant decrease in the vision, however, is the residue of some use	
Hearing impairment	Moderate hearing loss: hearing threshold level in the better ear is 41–60 dBHTL, not able to hear and repeat words spoken in normal voice at 1 m	17% of adult (USA)	+	++	(+)	+++	++	(34, 35)
	Severe hearing loss: hearing threshold level in the better ear is 61–80 dBHTL: not able to hear and repeat words using raised voice at 1 m	25% in 65–75 years	
	Profound hearing loss: hearing threshold level in the better ear is 81 dBHTL or more, not able to hear words when shouted into better ear	>70% in >75 years	
Malnutrition	No consensual definition. BMI can be used. Marker of malnutrition: involuntary loss of 5% of body weight over 1 month or 10% over 6 months	50% with BMI > 26 kg/m^2^ in 65–74 years	+(+)	+(+)	++	++(+)	++	(33, 36)
		Underweight (BMI < 21) 14% of females and 4% of males	
Osteoporosis	A disease characterized by low bone mass, microarchitectural deterioration of bone tissue leading to enhanced bone fragility, and a consequent increase in fracture risk + bone density measured with DXA, 2.5 standard deviations (SD) below the mean for healthy women aged 20–29 years, also referred to as a *T*-score of −2.5	Approximately 3.2% for entire population (USA) 14% in >50 years in Germany (24% in females)	+(+)	+(+)	++	++(+)	++(+)	(37 –39)

*ICD-10, International classification of diseases, World Health Organization*.

Table 2 describes the performances of the different tests found in the literature.

**Table 2:** **Tests for geriatric syndromes**.

Syndrome	Test	Performances	Time	Validated in PC	Validated in elderly	Reference
Cognitive impairment	Mini-COG	Se = 99%, Sp = 93%	2–5 min	X	X	(40)
	GPCOG	Se = 85, Sp = 86	2–5 min	X	X	(41)
	MIS	Se = 80, Sp = 96	2–5 min	X	X	(42)

Mood disorder	PHQ-9	Se = 77, Sp = 83 (any dep)	~1–2 min	X	X	(43)
	PHQ-2	Se = 82–86, Sp = 67–78 (any dep)	<1 min	X	X	(44)
	Two questions (similar to PHQ-2)	Se = 81–96, Sp = 51–72	<1 min			(45)
	GDS (15 questions)	Se = 81, Sp = 0.62		X	X	(46, 47)

Gait and balance impairment/falls	History of falls in past 1 year	Risk of fall next year: LR+ = 2.3–2.8	<1 min		X	(48)
	History of falls in past month	Risk of fall next year: LR+ = 3.8	<1 min		X	(48)
	Timed up and go	Extremely variable	1–2 min (?)			(49, 50)
	Tinetti test	Se = 80%, Sp = 87–89%	5 min (?)			(51)
	Stops walking When talking	Se = 77%, Sp = 68%	1–2 min (?)			(48)

Visual impairment	Questionnaire-based screening	Se = 90%, Sp = 44%		X		(52)
	Distance visual acuity (with presenting correction 20/40)	Se = 61%, Sp = 72%		X		(52)
	Snellen chart (distance)	Se = 74–94%, Sp = 87–89%			X	(53, 54)
	Snellen card (near vision)	Se = 77%, Sp = 68%			X	(54)

Hearing impairment	Whispered voice test	Median LR+ = 3.0–5.1 (several studies)	1 min (?)	X	X	(55, 56)
	Finger rub test	LR+ = 10 (CI 95% 2.6–43) (one single study)	<1 min			(55)
	Watch tick test	LR+ = 70 (CI 95% 4.4–1120) (one single study)	<1 min			(55)
	Single-item screening (for example, asking “Do you have difficulty with your hearing?”)	Median LR+ = 3.0–5.1 (several studies)	<1 min (?)			(55)
	Multiple-item patient questionnaire (for example, Hearing Handicap Inventory for the Elderly Screening Version)	Median LR+ = 3.0–5.1 (several studies)	5 min (?)	X	X	(55, 57)
	Handheld audiometer	Median LR+ = 5.8, Median LR− = 0.05, Se = 0.94–0.96, Sp = 0.69–0.70	5 min (?)	X	X	(55)
	Audioscope	Se = 94%, Sp = 72–90% (PC vs. specialist)	5 min (?)	X	X	(58)

Urinary incontinence	Two standardized questions	Se = 91%, Sp = 86%	1 min	X	X	(59)

Malnutrition	Mini Nutrition Assessment tool (MNA) short form	Se = 96, Sp = 98%	4–10 min		X	(60)
		Se = 98% Sp = 47–52% (>90 years)	4 min		X	(61)
	Simple screening tools (BMI and % loss of weight)	Validity (against dietitian assessment) of 61–92%	1 min		X	(62)

Osteoporosis	Low BMI, kyphosis/loss of height and fragility fracture	Any risk factor: Se = 68% Sp = 47% (NPV = 89%)		X	X	(63)
		All risk factors combined: Se = 60% Sp = 72% (NPV = 90%)	
	Wall-occiput distance (>0 cm)	Se = 60, Sp = 87	1 min	?	X	(64)
	Low weight (<51 kg)	Se = 22, Sp = 97	<1 min	?	X	
	Rib-pelvis distance (<2 fingers)	Se = 88, Sp = 46	<1 min	?	X	
	Tooth count (<20)	Se = 27, Sp = 92	<1 min	?	X	
	Self-reported humped back	Se = 21, Sp = 97	<1 min	?	X	

*?, unknown*.

The Data Sheet S2 in Supplementary Material describes the different tests developed for geriatric syndromes.

### Development of a BAT for General Practice

The Table 3 displays the BAT with screening tests for geriatric syndromes selected to be used by GPs.

**Table 3:** **Brief assessment tool for general practitioners**.

**Assessing the level of functional dependency (prior to screening for geriatric syndromes)**	**Four items of ADL or IADL** • Can you dress yourself? • Can you prepare your meals alone? • Can you make your own shopping? • Can you make your payment alone?	

**Syndrome**	**Test**	**Interpretation**

Cognitive impairment	Mini-Cog 1. Ask the patient to remember 3 words (insure that he/she retained them properly 2. Ask the patient to draw a clock with numbers and ask him/her to write 11h10 or 8h20 Instructions may be repeated but not other direction must be given 3. Ask the patient to repeat the 3 words	Clock: 2 points if the numbers are properly and time is correct, otherwise 0 points 3 words recall: 1 point/recalled word Interpretation 0–2 points: probable cognitive impairment 3–5 points: probable absence of cognitive impairment

Mood disorder	Two questions test 1. During the past month have you often been bothered by feeling down, depressed, or hopeless? 2. During the past month have you often been bothered by little interest or pleasure in doing things?	If one answer is “yes,” depression t suspected

Gait and balance impairment/falls	1 question Did you fall during the past year? Observation • How does the patient gets up from his chair • The standing balance (grabs a support, faltering, enlargement of the polygon) • His/her walk (gait symmetry, continuity, deviation from a path) • If he/she must stop waking when talking • How does the patient sits (drops back)	Increased risk of fait if “yes” to question

Visual impairment	Near vision Snellen pocket card	According to test’s results

Hearing impairment	Whisper test Whisper a question in each ear of the patient, standing back to him/her	Suspicion of hearing impairment if the patient can’t answer the question

Urinary incontinence	Four questions • Do you have difficulty holding urine or urge feelings? • Do you sometimes find it difficult to reach the toilet in time? • Do you have involuntary urine loss when coughing or effort? • Do you sometimes wear pads?	If one answer is “yes”: probable urinary incontinence

Malnutrition	Loss of weight >5% within 1 month, or >10% within 6 months	Present if positive

Osteoporosis	One question Did you loose height since you were 25 years old? Two measures • Distance wall-occipital • Distance ribs-pelvis	Increased risk of osteoporosis if lost of height >4 cm in women and >6 cm in men Wall-occipital: possible osteoporosis if >0 cm ribs-pelvis: possible osteoporosis if <2 fingers

*IADL, instrumental activities of daily living; ADL, activities of daily living*.

### Development of a Conceptual Framework and Integration of the BAT into General Practice

**Figure 1:**
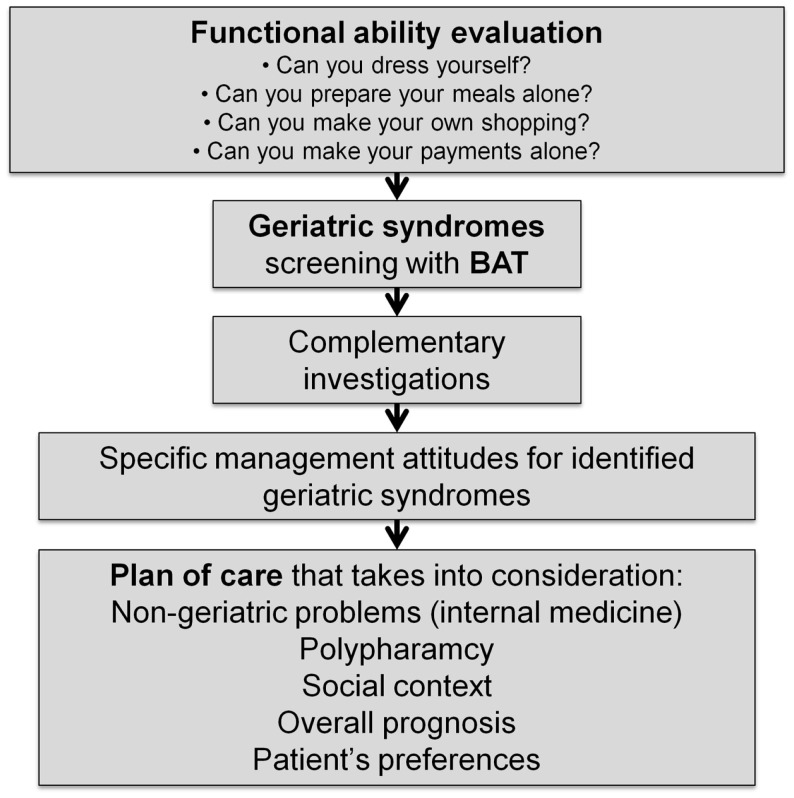
**Development of a conceptual framework for the integration of the screening and management of geriatric syndrome in general practice**.

Figure 1 describes the conceptual framework to integrate a geriatric comprehensive assessment of elderly patients into general practice. This figure shows that an assessment of functional ability is essential prior to any geriatric evaluation. It allows inquiry into patient function instead of beginning with pathological issues and helps to interpret the impact of geriatric or chronic health problems. Next follows the geriatric assessment itself (BAT), along with any eventual complementary investigations. Finally, all of this information is integrated into a plan of care that takes into consideration other key aspects: non-geriatric problems, polypharmacy, social context, overall prognosis, and patient’s preferences.

### Functional Ability Assessment

Functional performances should be routinely assessed in elderly patients in general practice, independently of the realization of a BAT. Thus, the first essential element that needs to be determined before to proceed to the screening of geriatric syndromes is the state of functional dependency of the patient. In that perspective, we did not include functional dependency as a geriatric syndrome but as an entry point to the assessment of the geriatric patient.

Few instruments have been developed to specifically assess functional dependency in the elderly. All rely on the measure of activities of daily life (ADL) to perform basic activities or instrumental activities. The two most well known are the score developed by Katz (or Katz index) for basic ADL that comprises six items (bathing, dressing, toileting, transferring, continence, and feeding) (65) and the Lawton IADL score that comprise eight items (phone, shopping, food preparation, housekeeping, laundry, mode of transportation, responsibility for own medication, and ability to handle financing) (66). Usually, instrumental activities are impaired prior to basic ADL (33, 67). Many combinations of these two instruments have been developed and assessed (68).

Surprisingly, no studies were found that assessed screening tools for functional decline in general practice. One study performed in general practice compared self-reporting of risk to standardized tools and concluded that reliability was high (69).

Because IADL items are lost first, we propose to keep four main basic and intermediate items of IADL as assessment of functional decline: dressing, cooking, shopping, and ability to handle financing. This choice is based on “what is lost first” in order to identify early enough patients with functional inability. Usually IADL items are more complex and usually first to be lost. Furthermore, there is a hierarchy in the loss of IADL, what guided our choice (70). One of the first IADL to be lost in dementia is handling financing, but not all patients (especially women) handle financing. Cooking is also early lost, but again, not all patients are preparing meals reason why we added a third item that is common to most patients, which is shopping (71). In regard to ADL, the choice was also made according to what is first lost among patients who can visit their GP. In brief, incontinence does not say a lot about overall dependence, a patient who cannot transfer, eat, toilet, or go alone to the toilet will most probably not be able to come to his GP, thus, remain mainly dressing alone.

### Timing for Performing a BAT Within General Practice

According to different recommendations such as the ACOVE guidelines (Assessing Care of the Vulnerable Elders) (20), a BAT should be performed once a year for each patient aged 70 years or over and as soon as a new health problem appears. This BAT should also be performed earlier in life if the patient suffers from multiple comorbidities.

## Discussion

This work describes the development of a comprehensive tool for the management of elderly patients in general practice. It encompasses the selection of the eight most relevant geriatric syndromes, the development of a brief geriatric assessment tool (BAT tool) for geriatric syndromes identification and a conceptual framework that integrates this geriatric assessment into routine general practice.

This work combines scientific evidence (prevalence of geriatric syndromes and their impact on functional decline) and a pragmatic approach to elderly care in general practice. Rather than proposing unachievable goals for elderly patients’ care in general practice (performing all the best screening tools for geriatric conditions identification and care), this work suggests an innovative way to combine geriatric assessment with the management of common chronic diseases.

One of the key elements of this integrative approach is its emphasis on the importance of assessing the functional ability of community-dwelling adults on a regular basis prior to systematically screening for geriatric syndromes. This is a crucial step as the measure of the level of functional dependency is essential in the perspective of a longitudinal follow-up of patients over time and provides physicians with a more global overview of the health status and its evolution over time. Furthermore, a better recognition of geriatric syndromes and functional status of patients in addition to existing comorbidities is likely to modify the health priorities and medication prescription that a physician sets in partnership with the patient. We can indeed postulate that for an old diabetic patient, it might be more important to enjoy good food and avoid malnutrition than having strict control of glycemia. Similarly, rigid control of hypertension often leads to orthostatic hypotension in elderly persons with an associated high risk of falls and secondary functional decline.

Within the described conceptual framework, polypharmacy is also seen in light of multimorbidity and functional impairment. Rather than only considering medication prescription according to the management of specific diseases, this framework forces the consideration of the potential impact of medications on other syndromes (i.e., anticholinergic drugs on cognitive performances).

To the best of our knowledge, this is one of the first attempts to comprehensively redesign the assessment of elderly patients in general practice rather than adding another layer of tests to usual practice. This work is therefore in line with recent work performed by the American Geriatric Society (AGS, “guiding principles for the care of older adults with multimorbidity” (72)) and adds the opportunity of integrating specific tools for the early diagnosis of geriatric syndromes. Indeed, and as mentioned in the introduction, most of the screening tools developed to date have simply been the transposition of tools developed in specialty setting and/or tested in general practice trial conditions.

This work contributes to design novel strategies for a high quality of care for elderly persons within the general practice context. Nevertheless, more work needs to be done to influence the quality of care in general practice setting. One component is that the education of health professionals working in GP practices should be reoriented toward the teaching of chronic diseases and geriatric syndromes management (73). Special attention should also be paid to make the screening tools proposed in this study available to GPs. For example, it could be integrated to continuous medical education or quality circles. In the frame of a cluster randomized trial that will be running on the implementation of this screening instrument, a specific training will be designed for GPs. The results of this trial will provide important information on the best way to make GPs using these tools.

Other external factors related to the system may also play a role such as the mode of remuneration. It is not yet defined how GPs can both provide good care for chronic diseases and simultaneously take into account the care of geriatric conditions. This requires rethinking the way medical consultations are performed for elderly in general practice, including the consideration of interdisciplinary approaches with the integration of other health professionals such as nurses or physical therapists. The present work might contribute to this reflection.

One of the weaknesses of this work is that it remains theoretical and needs to be tested in practice to assess if the tool can impact the quality of care of elderly patients. A study is already underway to assess the performance of the BAT in general practice. This study precedes a planned larger-scale cluster randomized trial that aims at investigating the benefits of this comprehensive tool when implemented into routine care. Another critique may be the selection of geriatric syndromes. Other geriatric conditions could have been included in the brief BAT tool (elder abuse, pressure ulcer, or sleep problems). However, the choice was made through explicit criteria such as prevalence, impact on functional decline, and QOL.

We believe, however, that the development of a more comprehensive, integrated, and concise screening tool for geriatric syndromes associated with a functional assessment of elderly patients and adequate management strategies has the potential to improve the quality of care to old patients in general practice.

## Author Contributions

NS and SM designed and performed together this work and designed the conceptual frame work. NS wrote the first draft of the manuscript.

## Conflict of Interest Statement

All authors declare having no conflict of interest. The study was internally funded by the institutions.
