# Supplementary material for: Development of a Comprehensive Approach for the Early Diagnosis of Geriatric Syndromes in General Practice
Source: Front Med (Lausanne). 2015 Nov 18;2:78. doi: 10.3389/fmed.2015.00078 (PMC4649036; doi:10.3389/fmed.2015.00078)
Supplement: Supplementary file 1 [file Data_Sheet_1.PDF]

Supplementary material 1

Development of a comprehensive approach for the early diagnosis of geriatric syndromes in general practice

Nicolas Senn[1*] MD PhD, Stéfanie Monod[2] MD

[1] Department of ambulatory care and community medicine (DACCM, Policlinique Médicale Universitaire), University of Lausanne, rue du Bugnon 44, 1011 Lausanne, Email : nicolas.senn@hospvd.ch

[2] Public Health Office, Canton de Vaud & CHUV, department of Medicine, rue des casernes 2, 1014 Lausanne, Email: Stefanie.Monod@vd.ch

* Corresponding author: Dr Nicolas Senn, Department of ambulatory care and community medicine (DACCM, Policlinique Médicale Universitaire), University of Lausanne, rue du Bugnon 44, 1011 Lausanne, Switzerland. Tel: +41 21 314 04 06, Fex: +41 21 314 88 88, Email : nicolas.senn@hospvd.ch

Rational and narrative report for selecting the geriatric syndromes

Cognitive impairment

There is a significant association between cognitive impairment and functional decline. Furthermore, dementia, with its behavioral complications, remains the main cause of institutionalization in the elderly.[1] Survival of patients with a diagnosis of dementia in primary care (PC) is much lower than that of a comparable population without the diagnosis.[2] Dementia is also associated with depression.[3]

Even if the effectiveness of procognitive treatments is modest clinically, many other interventions can be offered to patients, such as medication review and treatment of concomitant geriatric conditions.[4] Additionally, support to family and caregivers can improve the quality of life of demented patients and reduce their risk of morbidity and institutionalization.[5]

Mood disorder

Depression is significantly associated with functional decline, even after adjustment for confounding factors (comorbidities, cognitive status and alcohol consumption).[1] In addition, depression can worsen the prognosis of other comorbidities (cognitive disorders, cardiovascular diseases) which are frequently associated with depression.[6] On the other hand, it has been shown that treating depression in elderly can mitigate the risk of functional disability.[7]

Gait and balance impairment and falls

Gait impairment and falls are associated with increased mortality, morbidity, risk of functional decline and institutionalization.[8] Multifactorial interventions to improve mobility and reduce the risk of falls, such as the combination of exercise prescription, home hazards assessment, implementation of walking aids, and medication review, have been well documented in terms of efficiency.[9] [8]

Visual impairment

There is a significant association between visual impairment and functional dependency.[10] In addition, visual impairment has a direct impact on quality of life and is associated with an increased incidence of depression.[11] [12] Even if there is limited evidence on the direct benefits of assessing visual acuity in general practice, it should be part of a global assessment of elderly patients as it may indirectly reveal other problems such as errors in medication taking because of poor vision. A review of screening recommendations for visual impairment in general practice published in 2003 pointed

out the importance of performing systematic screening in old patients despite the lack of randomized trials to prove its effectiveness.[13]

Hearing loss

The direct link between hearing loss and functional dependence is weak. However, the association between hearing loss and depression and lower quality of life has been well documented.[14-16] It has also been shown that patients who receive hearing aid have an improved quality of life.[17]

Urinary incontinence

Urinary incontinence is associated with functional impairment, lower quality of life and decreased psychological well-being, as well as an increased risk of institutionalization.[18 19] The severity of incontinence seems to be more strongly associated with a lower quality of life than the type of incontinence.[20] Interventions to reduce urinary incontinence (lifestyle, behavioral, pharmacological and surgical) have been shown to be effective in improving urinary incontinence [21 22] Moreover, urinary incontinence renders older persons more vulnerable and prone to functional decline, and should therefore be part of a comprehensive evaluation.

Malnutrition

Few studies have provided good quality data on the association between BMI and functional decline.[23] One large study suggested an important relation between higher (> 30) and lower (<21) BMI in the elderly and functional decline.[24] Further, it has been shown that unintentional weight loss is strongly associated with functional decline.[25] It has also been shown that unintentional weight loss increased the risk of all cause mortality by 70% in old men, while intentional weight loss in the same cohort decreased the risk of all cause mortality by 41%.[26] More generally, it appears important to examine nutrition status and the related, potential treatable, physiological (acute/chronic illness, medication,…), psychological (depression) or social (low income, isolation) causes of malnutrition.[27]

Osteoporosis

Vertebral, hip and wrist fractures increase exponentially with age and are associated with functional decline.[28] More generally, lower skeletal and muscle mass are associated with functional decline.[29 30] As strategies to treat osteoporosis and prevent fractures exist, screening for osteoporosis is cost-effectives, especially in postmenopausal women.[31]

1. Stuck AE, Walthert JM, Nikolaus T, et al. Risk factors for functional status decline in community-living elderly people: a systematic literature review. Social science & medicine (1982) 1999;**48**(4):445-69.
2. Rait G, Walters K, Bottomley C, et al. Survival of people with clinical diagnosis of dementia in primary care: cohort study. BMJ 2010;**341**(aug05_2):c3584-.
3. Huang C-Q, Wang Z-R, Li Y-H, et al. Cognitive function and risk for depression in old age: a meta-analysis of published literature. International Psychogeriatrics 2011;**23**(04):516-25.
4. Spijker A, Vernooij-Dassen M, Vasse E, et al. Effectiveness of nonpharmacological interventions in delaying the institutionalization of patients with dementia: a meta-analysis. J Am Geriatr Soc 2008;**56**(6):1116-28.
5. Brodaty H, Green A, Koschera A. Meta-Analysis of Psychosocial Interventions for Caregivers of People with Dementia. Journal of the American Geriatrics Society 2003;**51**(5):657-64.
6. Bland P. Tackling anxiety and depression in older people in primary care. Practitioner 2012;**256**(1747):17-20, 2-3.
7. Lee Y, Park K. Does physical activity moderate the association between depressive symptoms and disability in older adults? International Journal of Geriatric Psychiatry 2008;**23**(3):249-56.
8. Society AG, Society G, Of AA, et al. Guideline for the Prevention of Falls in Older Persons. Journal of the American Geriatrics Society 2001;**49**(5):664-72.
9. Gillespie LD, Robertson MC, Gillespie WJ, et al. Interventions for preventing falls in older people living in the community. Cochrane database of systematic reviews (Online) 2012;**9**:CD007146.
10. Black AA, Wood JM, Lovie-Kitchin JE. Inferior visual field reductions are associated with poorer functional status among older adults with glaucoma. Ophthalmic and Physiological Optics 2011;**31**(3):283-91.
11. Mitchell J, Bradley C. Quality of life in age-related macular degeneration: a review of the literature. Health and Quality of Life Outcomes 2006;**4**(1):97.
12. Slakter JS, Stur M. Quality of life in patients with age-related macular degeneration: impact of the condition and benefits of treatment. Survey of ophthalmology 2005;**50**(3):263-73.
13. Yueh B SNMCHSPG. Screening and management of adult hearing loss in primary care: Scientific review. JAMA 2003;**289**(15):1976-85.
14. Boi R, Racca L, Cavallero A, et al. Hearing loss and depressive symptoms in elderly patients. Geriatr Gerontol Int 2012;**12**(3):440-5.
15. Bernabei V, Morini V, Moretti F, et al. Vision and hearing impairments are associated with depressive--anxiety syndrome in Italian elderly. Aging Ment Health 2011;**15**(4):467-74.
16. Cacciatore F, Napoli C, Abete P, et al. Quality of life determinants and hearing function in an elderly population: Osservatorio Geriatrico Campano Study Group. Gerontology 1999;**45**(6):323-8.
17. Mulrow CD, Aguilar C, Endicott JE, et al. Quality-of-life changes and hearing impairment. A randomized trial. Annals of Internal Medicine 1990;**113**(3):188-94.
18. DuBeau CE, Simon SE, Morris JN. The Effect of Urinary Incontinence on Quality of Life in Older Nursing Home Residents. Journal of the American Geriatrics Society 2006;**54**(9):1325-33.
19. Holroyd-Leduc JM, Mehta KM, Covinsky KE. Urinary Incontinence and Its Association with Death, Nursing Home Admission, and Functional Decline. Journal of the American Geriatrics Society 2004;**52**(5):712-18.
20. Barentsen J, Visser E, Hofstetter H, et al. Severity, not type, is the main predictor of decreased quality of life in elderly women with urinary incontinence: a population-based study as part of a randomized controlled trial in primary care. Health and Quality of Life Outcomes 2012;**10**(1):153.
21. Holroyd-Leduc Jm SSE. Management of urinary incontinence in women: Scientific review. JAMA 2004;**291**(8):986-95.
22. Swithinbank L, Hashim H, Abrams P. The effect of fluid intake on urinary symptoms in women. J Urol 2005;**174**(1):187-9.

23. Inzitari M, Doets E, Bartali B, et al. Nutrition in the age-related disablement process. J Nutr Health Aging 2011;**15**(8):599-604.
24. Larrieu S, Peres K, Letenneur L, et al. Relationship between body mass index and different domains of disability in older persons: the 3C study. Int J Obes Relat Metab Disord 2004;**28**(12):1555-60.
25. Ritchie CS, Locher JL, Roth DL, et al. Unintentional Weight Loss Predicts Decline in Activities of Daily Living Function and Life-Space Mobility Over 4 Years Among Community-Dwelling Older Adults. The Journals of Gerontology Series A: Biological Sciences and Medical Sciences 2008;**63**(1):67-75.
26. Wannamethee S SALL. REasons for intentional weight loss, unintentional weight loss, and mortality in older men. Archives of Internal Medicine 2005;**165**(9):1035-40.
27. Stajkovic S, Aitken EM, Holroyd-Leduc J. Unintentional weight loss in older adults. Canadian Medical Association Journal 2011;**183**(4):443-49.
28. Edwards BJ, Song J, Dunlop DD, et al. Functional decline after incident wrist fractures—Study of Osteoporotic Fractures: prospective cohort study. BMJ 2010;**341**.
29. Amigues I, Schott AM, Amine M, et al. Low skeletal muscle mass and risk of functional decline in elderly community-dwelling women: the prospective EPIDOS study. Journal of the American Medical Directors Association 2013;**14**(5):352-7.
30. Lui LY, Stone K, Cauley JA, et al. Bone loss predicts subsequent cognitive decline in older women: the study of osteoporotic fractures. J Am Geriatr Soc 2003;**51**(1):38-43.
31. Nayak S, Roberts MS, Greenspan SL. Cost-effectiveness of different screening strategies for osteoporosis in postmenopausal women. Annals of Internal Medicine 2011;**155**(11):751-61.
