# Supplementary material for: Development of a Comprehensive Approach for the Early Diagnosis of Geriatric Syndromes in General Practice
Source: Front Med (Lausanne). 2015 Nov 18;2:78. doi: 10.3389/fmed.2015.00078 (PMC4649036; doi:10.3389/fmed.2015.00078)
Supplement: Supplementary file 2 [file Data_Sheet_2.PDF]

Supplementary material 2

Development of a comprehensive approach for the early diagnosis of geriatric syndromes in general practice

Nicolas Senn[1*] MD PhD, Stéfanie Monod[2] MD

[1] Department of ambulatory care and community medicine (DACCM, Policlinique Médicale Universitaire), University of Lausanne, rue du Bugnon 44, 1011 Lausanne, Email : nicolas.senn@hospvd.ch

[2] Public Health Office, Canton de Vaud & CHUV, department of Medicine, rue des casernes 2, 1014 Lausanne, Email: Stefanie.Monod@vd.ch

* Corresponding author: Dr Nicolas Senn, Department of ambulatory care and community medicine (DACCM, Policlinique Médicale Universitaire), University of Lausanne, rue du Bugnon 44, 1011 Lausanne, Switzerland. Tel: +41 21 314 04 06, Fex: +41 21 314 88 88, Email : nicolas.senn@hospvd.ch

Tests for geriatric syndromes in general practice

Cognitive impairment

Several tests for identifying cognitive impairment are available and suitable for GPs. First, the Mini Cog, that combines a test of recent memory (three-item recall) and the clock drawing test is well-known and extensively studied, with good performances.(1) Second, the General Practitioner Assessment of Cognition (GPCOG), that also includes a clock drawing test and word recall, as well as a report of recent events, has slightly less good performances.(2) Finally, the Memory Impairment Screen (MIS) is also mentioned as suitable screening test for general practice. (3) Feasibility appears to be equivalent for these three tests but the performance of the Mini-cog is slightly better. Therefore, we proposed to keep this last test for cognitive impairment screening in general practice.

Mood disorder

Many screening test have been developed for depression in general practice.(4) Among the main tools described, there is the patient health questionnaire 9 (PHQ-9).(5, 6) It has been specifically assessed in elderly. A shorter version, the PHQ-2, was developed using the first two items of the PHQ-9. It has similarly high sensitivity and is very quick to perform.(7) Very similar to the PHQ-2 and even shorter, the two questions screening test, seems to be the most efficient screening tool for depression in general practice.(8) The questions are: 1) *"During the past month, have you often been bothered by feeling down, depressed, or hopeless?";* 2) *"During the past month, have you often been bothered by little interest or pleasure in doing things?".* Other tools such as the Geriatric Depression Scale (GDS) and its variations also performing well but are longer to administer.(4) We therefore proposed to keep the "2 questions" screening tool for the BAT.

Gait and balance disorder / falls

Little evidence exists on the best tools to be used in general practice. Most experts and studies agree however on the usefulness of a single question asking about falls within the last one month to one year, which has a good predictive value for the risk of falls during the next year.(9)

A popular test that was extensively studied to predict the risk of falls is the "Timed up and go test", where time to stand up, walk 3 meters, come back and sit down is measured. However results are mixed; a recent review found that the test may be helpful in a frail elderly population but not in a healthy elderly population.(10, 11) Moreover, instead of a quantitative test, we preferred to propose

an observation test for GPs. Observations of gait and balance can be conducted based on the Performance-Oriented Mobility Assessment test(12) and the dual-task test "Stops walking When talking". These tests have relatively good performance in predicting the risk of falls during the next six months. Instead of quantifying the number of falls, this observation identifies persons with gait and balance impairment even in the absence of past falls, and may further help the physician to complete a physical exam (neurological, osteo-articular, visual, etc).

In conclusion, we proposed an initial screening based on a single question about a past history of falls within the past year. If negative, we propose to proceed with a targeted gait and balance examination.(9)

Visual impairment

The simple Snellen test and the modified pocket card Snellen test are the two most widely recommended tests. We proposed therefore to keep the latter as it is easy to perform during a consultation. It has not, however, been properly assessed in general practice.(13, 14)

Hearing loss

Various tests can be used for screening: the whispered voice test, the finger rub test, the watch tick test, a single-item screening (*"Do you have difficulty with your hearing?"*), a multiple-item patient questionnaire (Hearing Handicap Inventory for the Elderly—Screening Version)(15) or a handheld audiometer (audioscope).(16) We proposed to use the whispered voice test, which includes a test of spoken voice and whispered voice, successively, at 30 and 60 cm from each ear. In several studies, it shows good negative predictive values with negative likelihood ratio (LR-) ranging from 0.03 to 0.5.(17, 18)

Urinary incontinence

Simple screening questions have been described as efficient for the identification of stress, urge or mixed urinary incontinency. Typically, both types can be identified by two questions: 1) Stress*: "during the past month, have you involuntarily been wetting yourself in connection with physical exertion (cough, sneezing, laughing)"?* 2) Urge*: "During the past month, have you experienced such a strong urge to pass water that it was impossible to get to the toilet in time?".(19)* Few formal investigations of performances of screening questions have been made in general practice. However,

it is widely recognized that these questions are self-explanatory and could be easily administered in general practice.(20)

Malnutrition

The Mini Nutrition Assessment -Short Form (MNA-SF) that encompasses 6 to 7 items is a validated tool for geriatric assessment of malnutrition.(21-24) Many other tools have been developed,(25) but a review of these instruments by Philips et al. identified the MNA-SF as the most well-suited screening tool for the elderly.(26) One study also revealed that MNA-SF administered by GPs identified both underweight and overweight patients. No studies have specifically investigated the performance of the MNA-SF in general practice, but the longer version (MNA) was assessed and proved to be efficient in this setting.(27, 28) However, because it is long to administer within a comprehensive geriatric assessment, it is unlikely to be feasible. Most tools incorporate an estimation of the patient's unintentional weight loss. A good marker of significant unintentional weight loss is a loss of >5% of usual weight in the past 3 years.(29) Finally, a simple score that includes BMI and percent of weight lost was validated against a full nutritional assessment.(30)

Considering the absence of a single validated test to identify malnutrition, we proposed to screen abnormal BMI and/or past history or observation (medical record) of unintentional weight loss (> 5% in one month or > 10% over 6 months).

Osteoporosis

Several clinical predictors of osteoporosis are described in the literature. Among the most frequently cited are height loss, wall-occiput and ribs-pelvis distance.(31) For example, a height loss greater than 4 cm in women (over the age of 25 years) is associated with a increased risk of vertebral fracture.(32) A non-zero distance wall-occiput and distance ribs-pelvis of less than two fingers were also associated with the presence of vertebral fractures.(33) Specific studies in general practice have also identified risk factors. One study by Versluis *et al* identified BMI, fragility fractures, and severe kyphosis and/or loss of height as good predictors of osteoporosis.(34) A review by Green et al. identified five physical examination maneuvers suggesting the presence of osteoporosis and/or spinal fracture: Wall-occiput distance (> 0 cm), low weight (< 51kg), rib-pelvis distance (< 2 fingers), tooth count (< 20) and self-reported humped back.(35) Among these signs, ribs-pelvis and wall-occiput distances have the highest sensitivity. We proposed therefore keeping both tests as screening clinical tests in the comprehensive assessment tool.

1. Bibliography

1. Borson S, Scanlan J, Brush M, Vitaliano P, Dokmak A. The Mini-Cog: a cognitive ‘vital signs’ measure for dementia screening in multi-lingual elderly. International Journal of Geriatric Psychiatry. 2000;15(11):1021-7. doi: 10.1002/1099-1166(200011)15:11<1021::aid-gps234>3.0.co;2-6.
2. Brodaty H, Pond D, Kemp NM, Luscombe G, Harding L, Berman K, et al. The GPCOG: A New Screening Test for Dementia Designed for General Practice. Journal of the American Geriatrics Society. 2002;50(3):530-4. doi: 10.1046/j.1532-5415.2002.50122.x.
3. Buschke H, Kuslansky G, Katz M, Stewart WF, Sliwinski MJ, Eckholdt HM, et al. Screening for dementia with the memory impairment screen. Neurology. 1999;52(2):231-8. PubMed PMID: 9932936.
4. Watson LC, Pignone MP. Screening accuracy for late-life depression in primary care: a systematic review. The Journal of family practice. 2003;52(12):956-64. PubMed PMID: 14653982.
5. Kroenke K, Spitzer RL, Williams JB. The PHQ-9: validity of a brief depression severity measure. Journal of general internal medicine. 2001;16(9):606-13. PubMed PMID: 11556941.
6. Phelan E, Williams B, Meeker K, Bonn K, Frederick J, Logerfo J, et al. A study of the diagnostic accuracy of the PHQ-9 in primary care elderly. BMC Family Practice. 2010;11:63. PubMed PMID: 20807445.
7. Arroll B, Goodyear-Smith F, Crengle S, Gunn J, Kerse N, Fishman T, et al. Validation of PHQ-2 and PHQ-9 to screen for major depression in the primary care population. Ann Fam Med. 2010;8(4):348-53. Epub 2010/07/21. doi: 10.1370/afm.1139. PubMed PMID: 20644190; PubMed Central PMCID: PMC2906530.
8. Whooley MA, Avins AL, Miranda J, Browner WS. Case-finding instruments for depression. Two questions are as good as many. Journal of general internal medicine. 1997;12(7):439-45. PubMed PMID: 9229283.
9. Ganz Da BYSPGRLZ. WIll my patient fall? JAMA. 2007;297(1):77-86. doi: 10.1001/jama.297.1.77.
10. Beauchet O, Fantino B, Allali G, Muir SW, Montero-Odasso M, Annweiler C. Timed Up and Go test and risk of falls in older adults: a systematic review. J Nutr Health Aging. 2011;15(10):933-8. PubMed PMID: 22159785.
11. Schoene D, Wu SMS, Mikolaizak AS, Menant JC, Smith ST, Delbaere K, et al. Discriminative Ability and Predictive Validity of the Timed Up and Go Test in Identifying Older People Who Fall: Systematic Review and Meta-Analysis. Journal of the American Geriatrics Society. 2013;61(2):202-8. doi: 10.1111/jgs.12106.
12. Tinetti ME, Williams TF, Mayewski R. Fall risk index for elderly patients based on number of chronic disabilities. The American journal of medicine. 1986;80(3):429-34. PubMed PMID: 3953620.
13. Ederer F, Krueger DE, Mowery RL, Connett J, Wentworth D. Lessons from the Visual Acuity Impairment Survey pilot study. American journal of public health. 1986;76(2):160-5. PubMed PMID: 3946697.
14. Woods RL, Tregear SJ, Mitchell RA. Screening for ophthalmic disease in older subjects using visual acuity and contrast sensitivity. Ophthalmology. 1998;105(12):2318-26. PubMed PMID: 9855166.
15. Yueh B SNMCHSPG. Screening and management of adult hearing loss in primary care: Scientific review. JAMA. 2003;289(15):1976-85. doi: 10.1001/jama.289.15.1976.
16. Lichtenstein MJ, Bess FH, Logan SA. Validation of screening tools for identifying hearing-impaired elderly in primary care. JAMA. 1988;259(19):2875-8. Epub 1988/05/20. PubMed PMID: 3285039.
17. Moyer VA. Screening for Hearing Loss in Older Adults: U.S. Preventive Services Task Force Recommendation Statement. Annals of Internal Medicine. 2012;157(9):655-61. doi: 10.7326/0003-4819-157-9-201211060-00526.
18. Pirozzo S, Papinczak T, Glasziou P. Whispered voice test for screening for hearing impairment in adults and children: systematic review. BMJ. 2003;327(7421):967. doi: 10.1136/bmj.327.7421.967.
19. Rohr G, Christensen K, Ulstrup K, Kragstrup J. Reproducibility and validity of simple questions to identify urinary incontinence in elderly women. Acta Obstetricia et Gynecologica Scandinavica. 2004;83(10):969-72. doi: 10.1111/j.0001-6349.2004.00557.x.

20. O'Neil B, Gilmour D. Approach to urinary incontinence in women. Diagnosis and management by family physicians. Canadian Family Physician. 2003;49(5):611-8.
21. Vellas B, Villars H, Abellan G, Soto ME, Rolland Y, Guigoz Y, et al. Overview of the MNA--Its history and challenges. J Nutr Health Aging. 2006;10(6):456-63; discussion 63-5. PubMed PMID: 17183418.
22. Guigoz Y. The Mini Nutritional Assessment (MNA) review of the literature--What does it tell us? J Nutr Health Aging. 2006;10(6):466-85.
23. Rubenstein LZ, Harker JO, Salvà A, Guigoz Y, Vellas B. Screening for Undernutrition in Geriatric Practice: Developing the Short-Form Mini-Nutritional Assessment (MNA-SF). The Journals of Gerontology Series A: Biological Sciences and Medical Sciences. 2001;56(6):M366-M72. doi: 10.1093/gerona/56.6.M366.
24. Kaiser MJ, Bauer JM, Ramsch C, Uter W, Guigoz Y, Cederholm T, et al. Validation of the Mini Nutritional Assessment short-form (MNA-SF): a practical tool for identification of nutritional status. J Nutr Health Aging. 2009;13(9):782-8.
25. Green SM, Watson R. Nutritional screening and assessment tools for older adults: literature review. Journal of Advanced Nursing. 2006;54(4):477-90. doi: 10.1111/j.1365-2648.2006.03841.x.
26. Phillips MB, Foley AL, Barnard R, Isenring EA, Miller MD. Nutritional screening in community-dwelling older adults: a systematic literature review. Asia Pac J Clin Nutr. 2010;19(3):440-9. Epub 2010/09/02. PubMed PMID: 20805090.
27. Calderon Reyes ME, Ibarra Ramirez F, Garcia J, Gomez Alonso C, Rodriguez-Orozco AR. [Compared nutritional assessment for older adults at family medicine settings]. Nutricion hospitalaria. 2010;25(4):669-75. Epub 2010/08/10. PubMed PMID: 20694306.
28. Beck AM, Ovesen L, Schroll M. A six months' prospective follow-up of 65+-y-old patients from general practice classified according to nutritional risk by the Mini Nutritional Assessment. Eur J Clin Nutr. 2001;55(11):1028-33.
29. Newman AB, Yanez D, Harris T, Duxbury A, Enright PL, Fried LP, et al. Weight Change in Old Age and its Association with Mortality. Journal of the American Geriatrics Society. 2001;49(10):1309-18. doi: 10.1046/j.1532-5415.2001.49258.x.
30. Laporte M, Villalon L, Thibodeau J, Payette H. Validity and reliability of simple nutrition screening tools adapted to the elderly population in healthcare facilities. J Nutr Health Aging. 2001;5(4):292-4. PubMed PMID: 11753498.
31. Krieg MA, Cuenot S, Lamy O. [Should we detect osteoporosis, and how?]. Rev Med Suisse. 2005;1(35):2248-50, 52-3. PubMed PMID: 16268446.
32. Vogt TM, Ross PD, Palermo L, Musliner T, Genant HK, Black D, et al. Vertebral fracture prevalence among women screened for the Fracture Intervention Trial and a simple clinical tool to screen for undiagnosed vertebral fractures. Fracture Intervention Trial Research Group. Mayo Clin Proc. 2000;75(9):888-96. PubMed PMID: 10994823.
33. Davis SR, Kirby C, Weekes A, Lanzafame A, Piterman L. Simplifying screening for osteoporosis in Australian primary care: the Prospective Screening for Osteoporosis; Australian Primary Care Evaluation of Clinical Tests (PROSPECT) study. Menopause. 2011;18(1):53-9 10.1097/gme.0b013e3181e77468.
34. Versluis RG, Papapoulos SE, de Bock GH, Zwinderman AH, Petri H, van de Ven CM, et al. Clinical risk factors as predictors of postmenopausal osteoporosis in general practice. The British journal of general practice : the journal of the Royal College of General Practitioners. 2001;51(471):806-10. PubMed PMID: 11677703.
35. Green Ad C-ECSBLDMTLKW. DOes this woman have osteoporosis? JAMA. 2004;292(23):2890-900. doi: 10.1001/jama.292.23.2890.
